# Diagnosis in Diseases

**Published:** 1890-03

**Authors:** B. F. Brittain


### DIAGNOSIS IN DISEASES.

BY B. F. BRITTAIN, M. D.

[Read before the Cherokee County Medical Society, and unanimously voted to Daniel'sTexas Medical Journal for publication.]

I ONCE thought I had the key to all the ills of the female sex. In 1856 I procured a copy of Bennet's work on the uterus. It was at that time a standard work on uterine pathology and the treatment of uterine disease. We can see the outcroppings of the teachings of Bennet on uterine diseases till the present day. He was a good, forcible writer, and left his impress on the physicians of the United States and his own country.

His writings and others of his notions have made it difficult often to diagnose the disease of the female. They drew attention so strongly to uterine inflammation that almost every symptom, whether neuralgia, cephalalgia, liver trouble, ovarian pain, irritable bladder, palpitation of the heart, and, in fact, all the neurotic symptoms were referred to uterine inflammation and ulceration. When we were called to a case presenting many of these protean symptoms we were taught to look to the womb for the cause and, if inflamed, especially the mucous membrane, it was said we would find as a sign of inflammation a mucous discharge from the os uteri. Well, in fact, this is partly so, but it is a great error to consider a mucous discharge from the womb a sign of uterine disease, for the mucous membrane of the uterus, like the schniderian membrane, secretes a mucus in the healthy condition.

Then let us bear in mind that before we diagnose inflammation of the endo-metrium, we must look for other signs and symptoms, than a mucus discharge alone, or even accompanied by pain in back, irritation of the bladder, etc.

We find in the writings of the present day a disposition to accuse the womb of the cause of very many of the nervous troubles of women. They invite our attention to the lacerated cervix or perhaps to the lacerated perineum as the cause of nervous troubles by reflex action. Now the fact is, fully one-third of the child bearing women have a lacerated cervix, and not more than one woman out of a dozen with lacerated cervix can justly charge her illness to the laceration.

In making up our diagnosis of the diseases of the female it is well to recollect that she has a nervous system, heart, lungs, stomach, liver and bowels, etc., and that each of these may and often is, diseased independent of the uterus. I am not contending, however, that a woman cannot have reflex symptoms of these organs from a diseased womb, but we merely throw out the caution to carefully investigate all cases before jumping to the conclusion of uterine disaease.

